# Ligand-bound integrin αvβ6 internalisation and trafficking

**DOI:** 10.3389/fcell.2022.920303

**Published:** 2022-08-24

**Authors:** Amelia Meecham, Lauren C. Cutmore, Pantelitsa Protopapa, Lauren G. Rigby, John F. Marshall

**Affiliations:** ^1^ Centre for Tumour Biology, Barts Cancer Institute, Queen Mary University of London, London, United Kingdom; ^2^ University of California, San Diego, San Diego, CA, United States

**Keywords:** αvβ6, endocytosis, internalisation, trafficking, A20FMDV2

## Abstract

The integrin αvβ6 is expressed at low levels in most normal healthy tissue but is very often upregulated in a disease context including cancer and fibrosis. Integrins use endocytosis and trafficking as a means of regulating their surface expression and thus their functions, however little is known of how this process is regulated in the context of αvβ6. As αvβ6 is a major target for the development of therapeutics in cancer and fibrosis, understanding these dynamics is critical in the development of αvβ6-targeted therapies. Following development of a flow cytometry-based assay to measure ligand (A20FMDV2 or LAP)-bound αvβ6 endocytosis, an siRNA screen was performed to identify which genes were responsible for internalising αvβ6. These data identified 15 genes (DNM2, CBLB, DNM3, CBL, EEA1, CLTC, ARFGAP3, CAV1, CYTH2, CAV3, CAV2, IQSEC1, AP2M1, TSG101) which significantly decreased endocytosis, predominantly within dynamin-dependent pathways. Inhibition of these dynamin-dependent pathways significantly reduced αvβ6-dependent migration (αvβ6-specific migration was 547 ± 128 under control conditions, reduced to 225 ± 73 with clathrin inhibition, and 280 ± 51 with caveolin inhibition). Colocalization studies of αvβ6 with endosome markers revealed that up to 6 h post-internalisation of ligand, αvβ6 remains in Rab11-positive endosomes in a perinuclear location, with no evidence of αvβ6 degradation up to 48 h post exposure to A20FMDV2. Additionally, 60% of ligand-bound αvβ6 was recycled back to the surface by 6 h. With studies ongoing using conjugated A20FMDV2 to therapeutically target αvβ6 in cancer and fibrosis, these data have important implications. Binding of A20FMDV2 seemingly removes much of the αvβ6 from the cell membrane, and upon its recycling, a large fraction appears to still be in the ligand-bound state. While these results are observed with A20FMDV2, these data will be of value in the design of αvβ6-specific therapeutics and potentially the types of therapeutic load.

## Introduction

Integrins are a family of heterodimeric cell surface proteins which, by definition, integrate the outside and the inside of the cell (Hynes, 2002). They consist of one α and one β subunit, and together form 24 unique α/β integrin combinations in the human body (Barczyk et al., 2010). αvβ6 is an integrin expressed exclusively on epithelial cells, and is mostly absent in healthy, adult tissues (Breuss et al., 1995). In cancer and fibrosis, αvβ6 expression often increases, and its expression is associated with disease progression and poorer prognosis (Hynes, 2002; Saini et al., 2015; Niu and Li, 2017).

αvβ6 is upregulated on an estimated one third of all solid cancers (Saha, 2011) and thus is a promising differentially expressed cell-surface molecule for detection and treatment of such cancers (Saha et al., 2010). The expression of αvβ6 is generally associated with poorer prognosis through a multi-faceted pro-tumorigenic response, including through its activation of TGF-β1 (Goodwin and Jenkins, 2009). While αvβ6 has been subject to intensive research towards development of αvβ6-targeting therapies, much of its basic biology remains not fully understood, including its internalisation and trafficking dynamics.

Furthermore, understanding the dynamics of αvβ6 endocytosis is important to those who are developing small molecule inhibitors of integrin αvβ6, such as those mimicking the high affinity (1.7 nM K_D_; Saha et al., 2010) αvβ6 specific 20-mer sequence NAVPNLRGDLQVLAQKVART (DiCara et al., 2007) (referred to as A20FMDV2) (Meecham and Marshall, 2021). As therapeutics are heading towards the clinic for the treatment and imaging of fibrosis and cancer (Hausner et al., 2007; Hausner et al., 2009; Slack et al., 2016; Meecham and Marshall, 2021), it will be essential to understand what happens to the αvβ6 after the inhibitor binds.

Integrins have relatively long half-lives [12–24 h (Lobert et al., 2010; Dozynkiewicz et al., 2012; Huet-Calderwood et al., 2017)], and with minimal known post-translational regulation (Meecham and Marshall, 2019), other mechanisms regulate their function and expression. Cells utilise endocytosis, trafficking, and recycling pathways to control the availability of integrin populations at the cell surface (Bridgewater et al., 2012; De Franceschi et al., 2015; Moreno-Layseca et al., 2019) and in part, therefore, their function. These processes are particularly important to integrins as the spatiotemporal regulation is key to their function; for example, integrins forming focal adhesions at the leading edge of migrating cells (Ramsay et al., 2007). Additionally, endocytosis and recycling are used to regulate the amount of active vs. inactive integrin at the cell surface (Arjonen et al., 2012). Although the importance of integrin trafficking is becoming increasingly recognised it remains poorly characterised (De Franceschi et al., 2015; Moreno-Layseca et al., 2019). Disruption of αvβ6 endocytosis has been shown to significantly impact the efficiency of αvβ6-mediated migration and invasion *in vitro* (Ramsay et al., 2007). Ramsay et al. (2007) showed that migration of cells towards αvβ6 specific ligands (LAP) was reduced when endocytosis was inhibited. This is promising evidence for those who postulate that inhibiting αvβ6 endocytosis could serve as a novel therapeutic mechanism in diseases where αvβ6 plays a central role.

Clathrin mediated endocytosis (CME) is the best characterised route of endocytosis (Kaksonen and Roux, 2018) and has been implicated in the endocytosis of both active (Ezratty et al., 2009), and inactive (Teckchandani et al., 2009) integrins. When a cell-surface expressed integrin is destined for clathrin mediated endocytosis, small invaginations of the cell membrane form, which are reinforced by clathrin (Kanaseki and Kadota, 1969), a scaffold protein that polymerises with the vesicle membrane. This process is initiated by a number of regulatory proteins such as AP2, which will bind directly to integrin tails and recruit clathrin to the site of the forming vesicle (Kaksonen and Roux, 2018).

Integrins are also endocytosed in a clathrin-independent manner, via clathrin-independent carriers. The most well-known is dynamin-dependent caveolar endocytosis (Oh et al., 1998). Caveolae are lipid rafts formed from caveolin proteins, moulded from cholesterol rich parts of the cell membrane. Caveolin proteins are anchored to the cytoskeleton (Stahlhut and van Deurs, 2000) and internalisation of caveolae is initiated by disruption of actin fibres (Pelkmans et al., 2002). A role for caveolar endocytosis has been demonstrated in multiple integrins, including β1 integrins, αLβ2, and αvβ3 (Wickström et al., 2002; Upla et al., 2004; Fabbri et al., 2005; Shi and Sottile, 2008; Bass et al., 2011).

Previous work has established that αvβ6 endocytosis is mediated by both clathrin-dependent and clathrin-independent endocytosis (Berryman et al., 2005; Ramsay et al., 2007; John et al., 2020). Additionally, it is known that clathrin-mediated αvβ6 endocytosis is at least partially initiated by HAX-1 (Ramsay et al., 2007), however the full molecular regulation of αvβ6 endocytosis is not understood.

Following endocytosis, integrins enter an early endosome, and are either degraded (Rainero and Norman, 2013) or recycled back to the membrane. Integrins have a long half-life (Lobert et al., 2010; Dozynkiewicz et al., 2012; Huet-Calderwood et al., 2017), and the majority of integrin molecules are recycled back to the plasma membrane (Bretscher, 1989; 1992). Comparable to endocytosis, this process is controlled by a network of intracellular proteins able to recognise integrin cytoplasmic tails and determine the fate of the integrin, regulating integrin dependent cell functions (Pellinen et al., 2006; White et al., 2007; Caswell et al., 2009).

Recycling routes used by integrins are not unique to this family of heterodimers and have been relatively well characterised in the context of other receptors such as the Transferrin receptor (TnfR) (Goldenring, 2015; Cullen and Steinberg, 2018). The two primary routes are Rab4-dependent short loop recycling and Rab11-dependent long loop recycling (Roberts et al., 2001; Jones et al., 2006). Short loop recycling occurs in a Rab4-dependent manner and is named due its ability to recycle back to the membrane without passing though the perinuclear recycling compartment (PNRC). Long loop recycling refers to the recycling which occurs via the PNRC. This is also partly regulated by Rab21, which binds to the α subunit cytoplasmic tail, and directs early endosomes to the PNRC (Pellinen et al., 2006). Integrin recycling has been implicated in the regulation of cell migration, cytokinesis and ECM remodelling (Caswell et al., 2009). In αvβ6 expressing tumours, αvβ6 is expressed at the leading invading edge of the tumour (Bates, 2005), which is thought to be achieved by recycling αvβ6 from the retracting edge of cells to the leading edge of the cell, promoting migration and invasion into healthy tissues (Frittoli et al., 2014).

Rab5 positive early endosomes mature in to Rab7 positive late endosomes (unless directed into recycling endosomes). One function of late endosomes is to degrade the associated cargo (Cullen and Steinberg, 2018), achieved by interaction with lysosomes, either by fusion or “kiss and run” events (Luzio et al., 2022). Towards this, during the endosome maturation process, the pH of the endosomes becomes more acidic (Mellman et al., 1986). This is a fundamental process in order for the proteases and lipases to optimally function in the endosome and has been used experimentally to track the location of integrins on their pathway to degradation (Barriere and Lukacs, 2008). The fate of αvβ6 post endocytosis remains unknown, however recent work has suggested that ligand-bound αvβ6 in cells of the normal lung cell line NHBE, is degraded; thus following exposure of NHBE cells to saturating concentrations of A20FMDV2 or Latency Associated Peptide (LAP), αvβ6 expression significantly decreased and did not return to pre-exposure surface-levels for up to 50 h post-washout of the peptides (Slack et al., 2016).

The aim of the work described here is to establish the basic internalisation and trafficking dynamics of αvβ6 following ligand engagement, to identify the genes responsible for regulating αvβ6 endocytosis, and determine the role of endocytosis in αvβ6-specific functions, and post-endocytosis events including recycling, degradation, and signalling.

## Materials and methods

### Cell lines

A375 and A375β6 cell lines were previously developed in-house (DiCara et al., 2007). C76, C139 and C102 are circulating tumour cell (CTC)-derived PDAC cell cultures also developed in house, and characterised by Raj et al., 2021 (Raj et al., 2021). All cell lines were grown as adherent monolayers under standard cell culture conditions (5% (volume/volume) of carbon dioxide (CO2)/air at 37°C) in either Dulbecco’s minimum essential medium (DMEM) (D6429, Gibco) (A375, A375β6, MDAMB468, MCF10A, MCF10ACA1α and BT20) or Roswell Park Memorial Institue-1640 medium (RPMI) (R8758, Gibco) (CTC cells, H358, H441 and H322M) supplemented with 10% Foetal Bovine Serum (10500-064, Gibco). NHBE cells were cultured using Bronchial Epithelial Cell Growth Medium (BEBM) supplemented with BEGM BulletKit Growth Factors (CC-4175, Lonza) and HPDE with Keratinocyte serum free media supplemented with Keratinocyte SFM kit (17005042, ThermoFisher). Integrin αvβ6 expression levels in these cell lines can be found in Supplementary Table 1.

### Fluorochrome-labelled peptides

A series of fluorochrome-labelled variants of A20FMDV2 were synthesised by Peptide Protein Research Ltd. (Cambridge, United Kingdom). The original A20FMDV2 peptide was generated with a Lys (biotinylated) substituted for Ala in position 2 (NK(biotinyl)VPNLRGDLQVLAQKVART) and extended on its N-terminus by a GSGSGSGSGS [(GS)_5_] linker and a terminal cysteine. The fluorochromes Cy3 or Cy5 were conjugated directly to the N-terminus to create Cy3-bioA20FMDV2 and Cy5-bioA20FMDV2, respectively.

A separate scrambled peptide (NK(biotinyl)LRDQTGLKNPVQLARAV) was also extended by a (GS)_5_ spacer and labelled on the N-terminus with Cy3 to create Cy3-bioA20ran. In order to monitor the degree of internalisation from the surface, a variant that had a TCEP cleavable di-sulphide bond between the linker and the Cy3/Cy5 (Cy3/5-SS-(GS)5-NK(bio)VPNLRGDLQVLAQKVART) was synthesised and called Cy3/5-SS-bioA20FMDV2. All peptides were >95% pure by HPLC (data not shown). Additionally, A20FMDV2-pHrodo was generated by conjugating via di-sulphide bond a pH-sensitive pHrodo green maleimide dye (P35370, ThermoFisher) to an A20FMDV2 peptide featuring a terminal cysteine (NK(Bio)VPNLRGDLQVLAQKVARTC).

### Flow cytometry

Briefly, cell monolayers were dissociated using Trypsin-EDTA (L11-004, Gibco), and diluted in DMEM to 2 × 10^5^ cells for each condition. Cells were incubated with 100 nM of peptide on ice for 60 min. The αvβ6 blocking antibody 264RAD was used at 10 μg/ml. For unconjugated peptides and antibodies, appropriate secondary AlexaFluor antibodies were used. 10,000 events were acquired using BD LSRFortessa flow cytometer equipped with 488 nm blue, 640 nm red, 405 nm violet and 561 nm yellow-green lasers (BD Biosciences^©^, Becton, Dickinson & Co.).

### Fluorescence microscopy

3 × 10^4^ cells were prepared on 13 mm diameter glass coverslips, and subsequently fixed in 4% paraformaldehyde (P6148, Sigma Aldrich ^®^Co. LLC). To look also at intracellular expression of proteins, cells were permeabilised with 0.1% TritonX-100 in PBS (AAA16046AE, Alfa Aesar^©^ ThermoFisher) prior to antibody or peptide staining. For cell surface staining only, this step was performed following primary and secondary antibody staining.

Primary antibodies [62OW (anti human β6 subunit-developed in house) 10 μg/ml, EEA1 (sc-137130, SantaCruz) 1:100, LAMP1 (ab62562, Abcam) 1:2,000], were prepared in DMEM 0.1/0.1 buffer [0.1% (w/v) bovine serum albumin (BSA) (A7906, Sigma Aldrich)/0.1% (w/v) sodium azide (NaN3) (S8032, Sigma Aldrich)], and incubated for 1 hour at RT. Following three washes with DMEM 0.1/0.1, appropriate secondary antibody was added and incubated for 30 min at room temperature (RT) in the dark. Peptides were used at 500 nM. Cells were counterstained with DAPI (62248, ThermoFisher) (1:5,000) and Phalloidin-Rhodamine (R415, ThermoFisher) (1:1,000) for 10 min at RT. Subsequently, coverslips were mounted using Mowiol (81381, Sigma) and left to dry overnight. Image acquisition was performed using Zeiss LSM710 or LSM880 confocal microscopes (Barts Cancer Institute Microscopy Core Facility, QMUL, London) using Zeiss Zen imaging software (Carl Zeiss®, AG). Samples were imaged using a 63x Apochromat/1.4 NA Oil immersion objective. Image processing was performed using ImageJ (ImageJ64 v1.46r, National Institutes of Health).

### Internalisation assays

#### Flow cytometry

**FIGURE 1:**
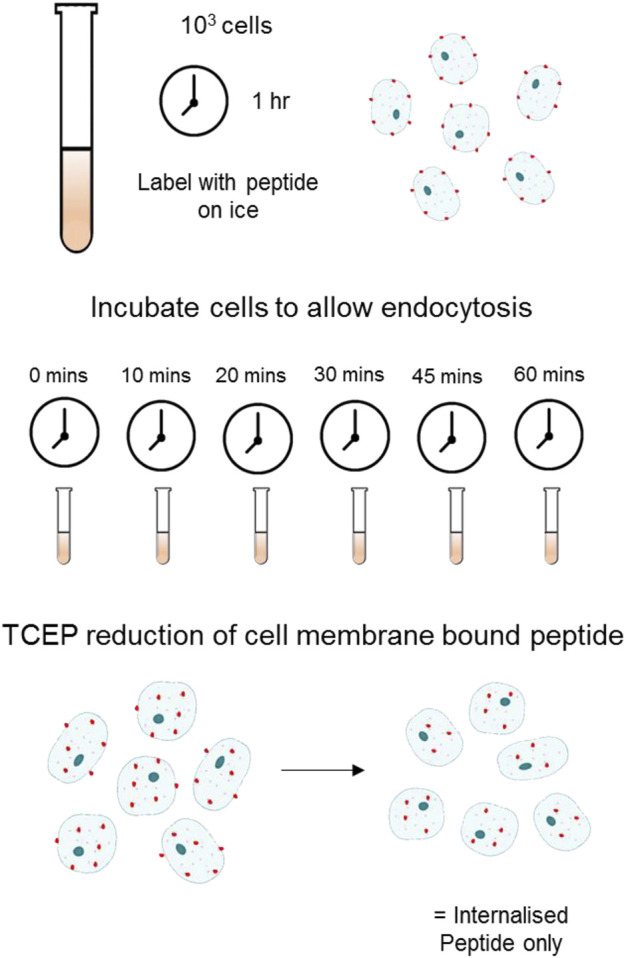
Schematic of Flow Cytometry Internalisation Assay using TCEP Reduction Cells labelled with a fluorescently tagged peptide were incubated at 37°C in appropriate cell culture medium + FBS for the indicated time allowing for internalisation. Following endocytosis and washing, 100 mM of TCEP was added to each sample to cleave the fluorochrome selectively from the surface bound pool of peptide.

Cells were prepared as described above for flow cytometry. Following staining with peptide, cells were placed at 37°C for the indicated time to allow for internalisation. To remove the fluorescent cyanine dyes from surface bound A20FMDV2, cells were treated with 100 mM TCEP (tris(2-carboxyethyl)phosphine) (T2256, ThermoFisher), to reduce the disulphide bond between the N-terminus of the peptide and the attached cyanine dye (Figure 1). Internalisation at each time point was calculated as the number of fluorescent cells following reduction with TCEP. For recycling experiments, TCEP treated cells were returned to 37°C for various periods of time (up to 6 h) and then re-exposed to TCEP to remove any surface re-exposed αvβ6 still bound to fluorochrome. To calculate the amount of surface re-expression of ligand-bound αvβ6, the difference in the cellular fluorescence before and after this second TCEP treatment was calculated.

#### Image stream

1 million cells in 60 µl of DMEM were prepared as described above for flow cytometry. 10,000 events were acquired on the Amnis^®^ ImageStream^®^X Mk II Imaging Flow Cytometer (Luminex). Acquisition was performed following gating of cells based on size and those in focus. Analysis was performed using the IDEAS^®^ software (Amnis). A mask was created using the “Erode” function and used to determine the boundary between the inside and outside of the cell, visualising cells using brightfield imaging. Internalisation was quantified as the ratio of fluorescence inside of the mask area against total cell fluorescence.

#### Fluorescence microscopy

Cells were prepared on coverslips as described. Following staining with the indicated peptide, the 24-well plate containing coverslips was returned to 37°C for the indicated time in appropriate growth media to allow for internalisation. Cells were placed immediately on ice and fixed for 10 min in paraformaldehyde (P6148, Sigma Aldrich Co. LLC) following incubation. Counterstaining was performed as described above.

### Western blotting

Lysates were prepared using RIPA buffer (50 mM TrisHCl pH 8, 150 mM NaCl, 1 mM EDTA, 0.1% SDS, 1% Nonidet-P40) containing protease and phosphatase inhibitors (1:100) (Calbiochem; 539131 and 524625, respectively) and quantified using commercially available colorimetric assay (Bio-Rad Protein Assay Kit; 5000112). Equal protein amounts were loaded in to wells of mini-SDS-PAGE gels and run at 80 V for 30 min, followed by 90 min at 120 V. Resolved proteins were transferred onto a nitrocellulose membrane (Amersham Hybond TM ECLTM RPN303D, GE Healthcare^©^) by wet transfer using BioRad Mini-PROTEAN^®^ Trans-Blot^®^ Module (1658029, Bio-Rad Laboratories Inc.) for 1 h at 120 V. The blots were probed overnight with primary antibody in blocking buffer at 4°C. αvβ6 was detected using goat polyclonal antibody C19 (sc-6632**,** SantaCruz, 0.2 μg/ml).

### Migration assay

Cell migration assays were performed using polycarbonate cell culture inserts (8 µm-pore size, 24 well Thinsert, 662638, Greiner). Inserts were coated on their underside with either LAP (0.5 μg/ml, L3408, Sigma Aldrich), or Fibronectin (10 μg/ml, F2006, Sigma Aldrich) in 50 µl PBS. After 1 h at RT, the solution was removed, the Transwell washed with PBS, and returned to a 24 well plate. 1 × 10^5^ cells were added to the top of the Transwell and incubated for 16 h at 37°C. Transwells were washed twice in PBS and fixed in 4% formaldehyde. Following a further wash in PBS, the Transwells were placed in Crystal Violet dissolved in 70% methanol. Transwells were washed three times in PBS and allowed to dry overnight. The following day, the upper side of the membrane of the Transwells was washed using a damp cotton bud to remove any cells adhered. Cells adhered to the bottom of the transwell were visualised and quantified using light microscopy.

### siRNA transfections

Cells prepared to 30%–40% confluency were transfected with Dharmacon siRNA smartpools. Brieflly, 50 mM of siRNA smartpool was added dropwise to cells combined with Interferrin (Polyplus Transfections) as per the manufacturer’s instructions. At 72 h post transfections, cells were harvested for downstream experiments.

### Statistical analysis

Quantitative data are presented as mean ± standard deviation, unless otherwise stated. Appropriate statistical tests (dependent on normality of distribution, variance, data type and the number of conditions) were performed in Prism v8 (Graphpad Software), and significance was defined as *p* < 0.05, *p* < 0.01 and *p* < 0.001.

## Results

### Establishing the rate of αvβ6 endocytosis

**FIGURE 2:**
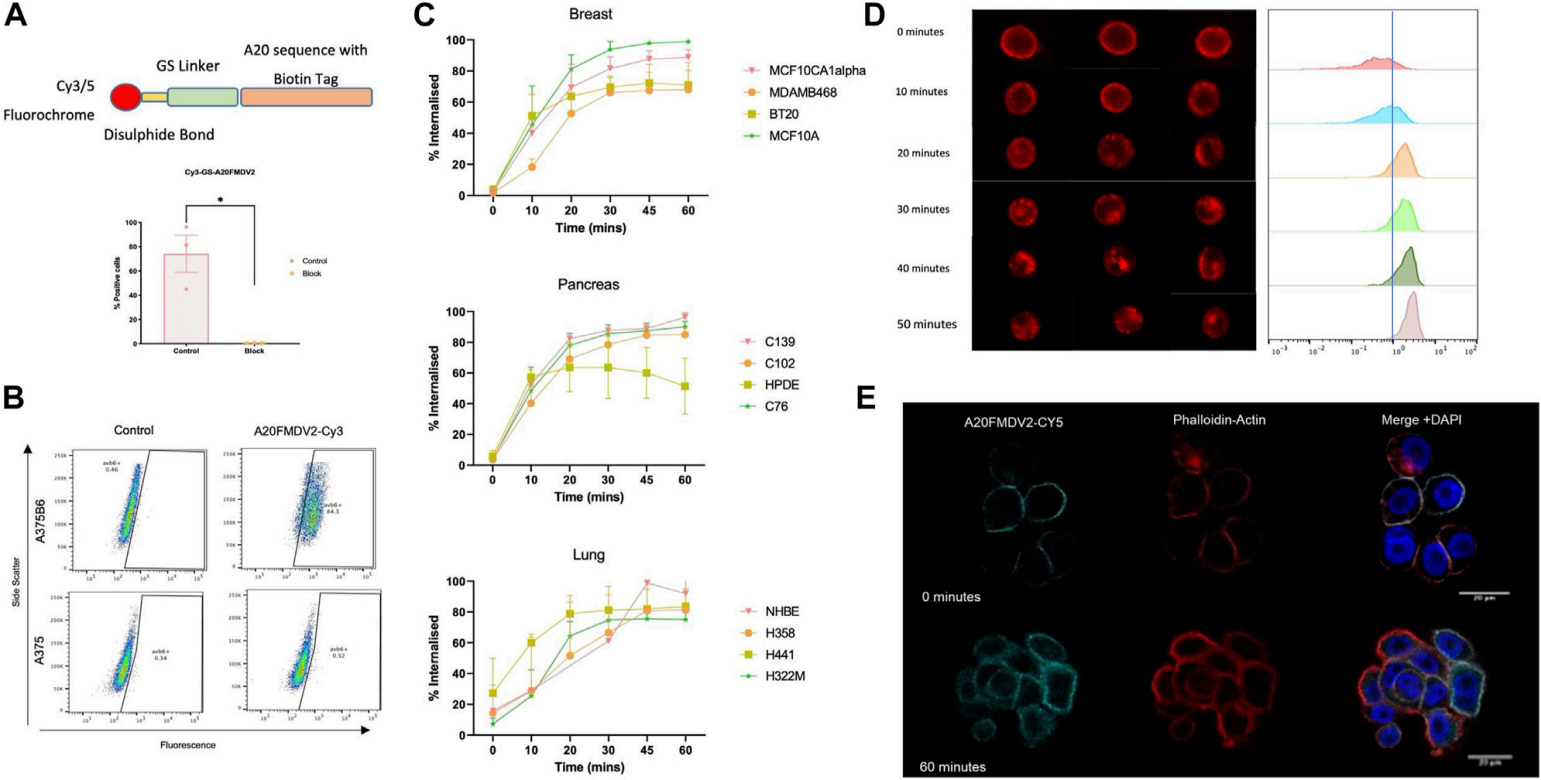
αvβ6 bound A20FMDV2 is internalised into cells over 60 min. **(A**, Top**)** Design of the Cy3/5-SS-(GS)_5_-bioA20FMDV2 featuring a disulphide bond between the fluorochrome and GS linker, to allow for cleavage of the fluorochrome only. (Bottom) Pre-treating A375PB6 cells with αvβ6 specific antibody 53A2 significantly reduced binding of Cy3-SS-(GS)_5_-bioA20FMDV2 (74.1 ± 26.4% to 0.44 ± 0.17%, *p* < 0.05), confirming it retained specificity. Data represents live single cells, mean ± SD, *n* = 3, paired **(B)** A375P and A375PB6 cell lines were exposed to 100 nM of Cy3-SS-(GS)_5_-bioA20FMDV2 peptide for 1 hour, and binding quantified by flow cytometry, using Cy3-bioA20ran to determine the non-specific binding and gate the positive fraction. **(C)** Internalisation of Cy5-SS-(GS)_5_-bioA20FMDV2 was determined in a range of cell types of breast, pancreas and lung origin. Each panel consisted of both normal and cancer cell lines. No Significant differences were observed in the rate of internalisation between cell types. In each cell line, at least 50% of peptide was internalised in 30 min. **(D)** A20FMDV2 internalisation quantification using *ImageStream* flow cytometer. (Left) representative images of cells at each time point revealing that Cy5-(GS)_5_-bioA20FMDV2 is internalised from the cell membrane from 10 min of incubation at 37°C. (Right) Quantification of the relative internalisation shows that the proportion of Cy5-(GS)_5_-bioA20FMDV2 inside the cells at each time point is 0 min = 11.5%; 10 min = 27.8%; 20 min = 74.1%; 30 min = 76.7%; 40 min = 84.4%; 50 min = 94.6%. Data represents single live cells only. **(E)** Immunofluorescent staining of Cy5-(GS)_5_-bioA20FMDV2- αvβ6 endocytosis. αvβ6 bound A20FMDV2 is exclusively localised at the cell membrane at 0 min, while after 60 min, αvβ6 can be seen inside the cell. Images were acquired using LSM880 confocal microscope using a ×63 objective. Cy5-(GS)_5_-bioA20FMDV2 (Cyan), DAPI (blue) and Phalloidin (Red).

A fluorescently conjugated biotinylated A20FMDV2 peptide was generated featuring a glycine-serine (GSGSGSGSGS) linker and disulphide bond between the fluorochrome and the A20FMDV2 αvβ6-specific sequence (Figure 2A) (Cy5-SS-(GS)_5_-bioA20FMDV2). The peptide retained specificity for αvβ6 (Figure 2B) even with the addition of these features and peptide-binding was inhibited using the αvβ6 blocking antibody 264RAD (Figure 2A). Using Cy5-SS-(GS)_5_-bioA20FMDV2, ligand-bound αvβ6 endocytosis was measured using flow cytometry (Figure 2C) in a range of cell lines. Surface bound peptide was removed selectively using the reducing agent TCEP, with the remaining fluorescence representing internalised A20FMDV2. No significant difference in peptide internalisation was observed when the cells were in suspension compared to adhered (Supplementary Figure 1).

Breast, pancreas, and lung cancer cell lines demonstrated significant internalisation of A20FMDV2 over the course of 60 min, with 50% of the peptide internalised after 30 min in all 12 cell lines. No differences in the rate of internalisation were observed between tissue types, or in normal vs. cancer cells. Internalisation of Cy5-(GS)_5_-bioA20FMDV2 was also confirmed using an ImageStream flow cytometer (Figure 2D) and immunofluorescence microscopy (Figure 2E). Quantification of images obtained from the image stream revealed that 94.6% of the peptide was inside the cell after 50 min.

### Determining the molecular regulation of αvβ6 endocytosis

**FIGURE 3:**
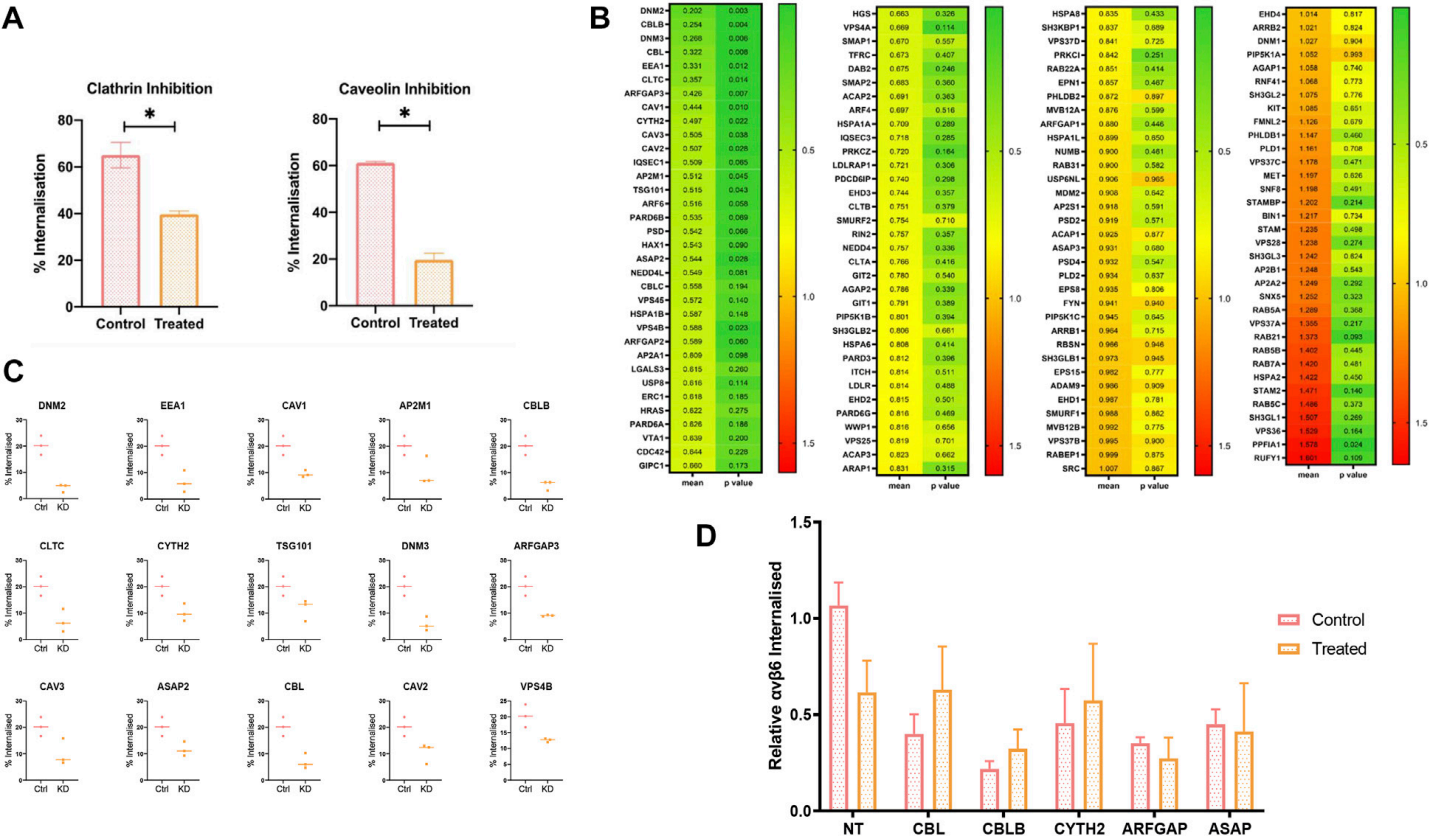
An siRNA screen to determine molecular regulators of A20FMDV2-αvβ6 endocytosis. **(A)** C76 cells were pre-treated with either 2 ug/ml of Chlorpromazine (clathrin inhibitor) or 10 ug/ml of Filipin (Caveolin inhibitor) before performing A20FMDV2 internalisation assay as described. Both Clathrin and Caveolin inhibition resulted in a significant decrease (*p* < 0.05) in the rate of internalization (65 ± 9.5% to 39 ± 2.5% and 61 ± 0.8% to 19.7 ± 3.9%, respectively) (*n* = 3). **(B)** Cherry-picked siRNA library featuring 140 gene pools was transfected in to C76 cells. 72 h post transfection an internalisation assay by flow cytometry was performed as previously described. The first column of the heat map represents the relative internalisation compared to the non-targeting, with green representing a decrease and red indicating an increase. The second column represents the *p*-value, with green representing a lower *p* value, and red a higher *p* value. Data represents mean values (*n* = 4). **(C)** Fifteen genes are shown from the flow cytometry screen which significantly reduced the rate of αvβ6-A02FMDV2 endocytosis in to C76 cells (*p* < 0.05). Data represents mean ± SD. *p* < 0.05, as determined by student’s *t*-test. **(D)** C76 cells were transfected with 50 mM of the indicated siRNA smartpool, 72 h later the cells were either treated with Clathrin inhibitor or vehicle control, and an internalisation assay performed as previously described using A20FMDV2. Again, compared with the non-targeting controls KD of each gene resulted in significant reduction in internalisation (NT vs. CBL: *p* = 0.01; CBLB: *p* = 0.002; CYTH2: *p* = 0.04; ARFGAP: *p* = 0.019; ASAP: *p* = 0.0125). However, there was no significant change in internalisation with the addition of Clathrin inhibitor, compared to the siRNA knockdown alone [NT 24 ± 4.7% vs. 14 ± 6.5% (*p* = 0.09), CBL 9 ± 4% vs. 14 ± 8%, CBLB 5 ± 1.6% vs. 7.4 ± 3.9%, CYTH2 10 ± 7% vs. 13 ± 11.7%, ARFGAP 8 ± 1% vs. 6.2 ± 3.5%, ASAP 10.3 ± 3.1% vs. 9.4 ± 9.9%] (*n* = 3). Data suggest that these five genes operate in the clathrin-mediated pathway.

Small molecule inhibitors Chlorpromazine (Wang et al., 1993) and Filipin (Schnitzer et al., 1994) were used to specifically inhibit clathrin and caveolin internalisation pathways, respectively, in the pancreatic cancer cell line C76 (Figure 3A). Cells treated with Chlorpromazine internalised significantly less αvβ6-bound Cy5-SS-(GS)_5_-bioA20FMDV2 after 20 min compared to the control (39 ± 2.5% vs. 65 ± 9.5%, *p* < 0.05). A significant reduction in internalisation was also observed in caveolin inhibited cells, with a 41% reduction in the number of cells with internalised αVβ6-A20FMDV2 (from 61 ± 0.8% to 19.7 ± 3.9%, *p* < 0.05).

To investigate this further, a panel of siRNA pools targeting 135 genes known to be involved in endocytosis were selected for transfection into C76 cells in order to characterise ligand-bound αvβ6 endocytosis. These included genes involved in clathrin and caveolin mediated endocytosis, and additionally genes involved in dynamin-independent pathways (Figure 3B).

The percentage of internalised A20FMDV2 in each knockdown condition was quantified and normalised to that of the non-targeting control transfected cells (mean internalisation 23.6 ± 5.4% after 20 min). These mean values (*n* = 4) can be found in Figure 3B, sorted by the knockdown which had the biggest reduction in internalisation, DNM2 (4.19 ± 1.25%), equivalent to an 80% reduction in endocytosis compared to the non-targeting control. The biggest increase in endocytosis was induced by the RUFY1 knockdown (41.5 ± 10.7%), which is equivalent to a 60% increase in the rate of endocytosis. A paired *t*-test revealed that 16/135 of the genes knocked-down resulted in a significant change in the amount of endocytosis. Fifteen of these significantly decreased the rate of endocytosis (*p* < 0.05) (Figure 3C), while one gene increased the rate of endocytosis.

Two of the genes significantly reducing αvβ6 endocytosis (CLTC and AP2M1) are exclusive regulators of clathrin-mediated endocytosis (CME) (Kaksonen and Roux, 2018), while CAV1, CAV2 and CAV3, are exclusively involved in caveolin mediated endocytosis (Lajoie and Nabi, 2007; Mayor and Pagano, 2022), confirming the role of both clathrin and caveolin in the internalisation of ligand bound αvβ6.

The remaining genes which significantly reduced αvβ6 endocytosis were involved in overlapping endocytosis pathways. Crucially, five of these genes (CBL, CBLB, CYTH2, ARFGAP3 and ASAP2) have previously been identified to play a role in dynamin-independent internalisation in addition to CME. To identify a potential role for these genes independent of clathrin, knockdown was repeated, and the internalisation assay performed in the presence or absence of the clathrin inhibitor, chlorpromazine (Figure 3D). Comparison of the rate of internalisation in the untreated KD cells compared to the drug-treated KD cells would therefore indicate whether the gene of interest is acting to reduce αvβ6 internalisation via clathrin alone, or, in addition, by another endocytosis pathway.

There was a reduction in internalisation in the cells transfected with non-targeting (NT) siRNA following inhibition with chlorpromazine, however this was not significant (*p* = 0.06) (24 ± 4.7% vs. 14 ± 6.5%) (Figure 3D). No significant differences were observed between any of the treated- vs. untreated-cells in any knockdown condition, suggesting that these genes are regulating endocytosis in a clathrin-dependent manner, with no additional reductions in the rate of endocytosis obsevered independent of clathrin.

While efficient knockdown was confirmed for only some genes used in the panel (Supplementary Figure 4), due to the high number of hits involved in previously confirmed internalisation pathways significantly reducing endocytosis (clathrin and caveolin mediated), we were confident in the screen’s accuracy. However, we do acknowledge the potential of false negatives in the screen.

### Functional implications of inhibiting αvβ6 endocytosis

**FIGURE 4:**
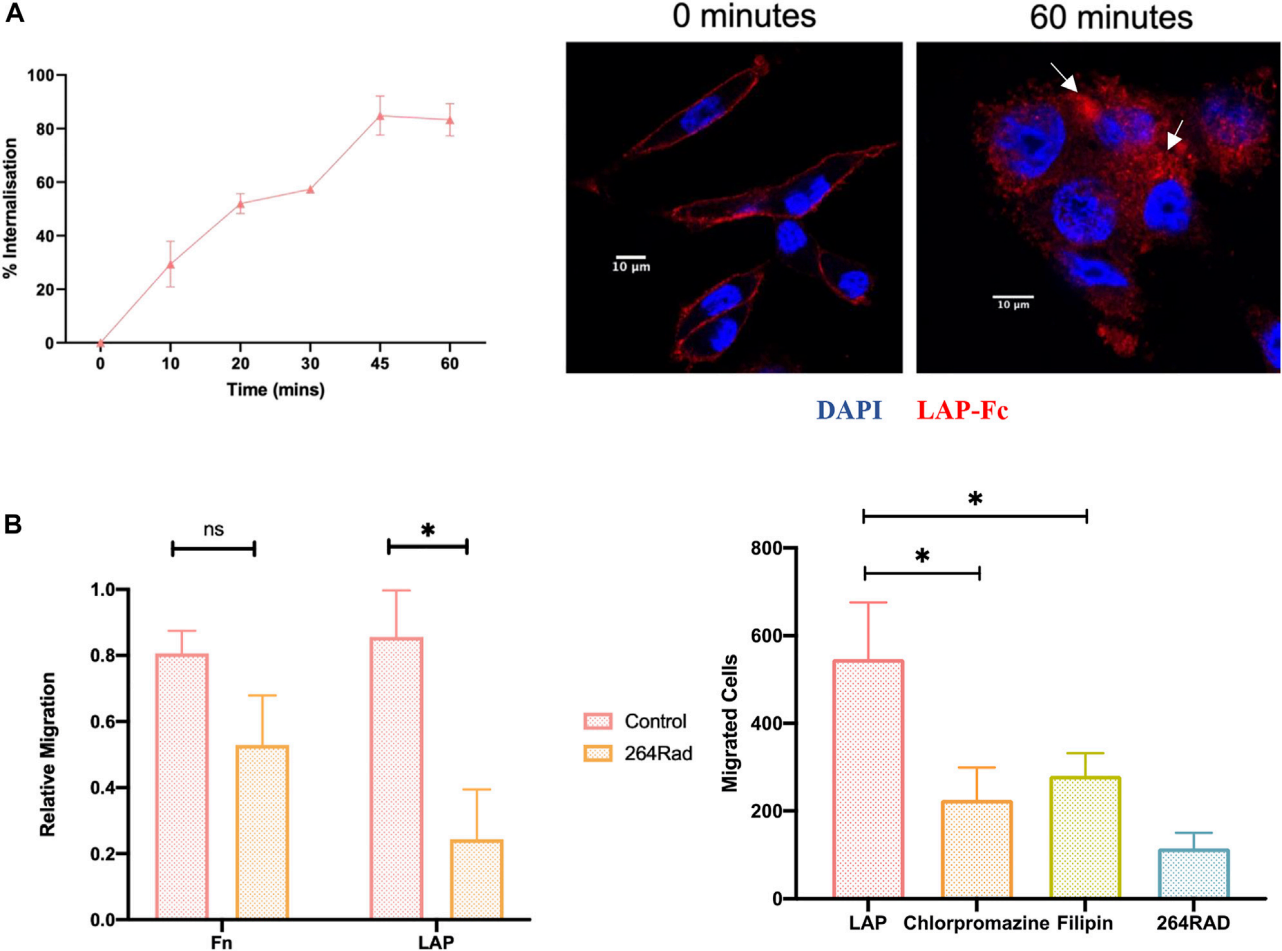
Inhibition of ligand-bound αvβ6 endocytosis reduces the rate of cell migration. **(A)** Internalisation of LAP in C76 cells. (Left) Using flow cytometry, C76 internalised 83 ± 10% of LAP-Fc in 60 min Data represents mean ± SD (*n* = 3). (Right) LAP-Fc internalisation was also assessed using immunofluorescent microscopy, again revealing internalisation of LAP-Fc (red) inside the cell after 60 min. **(B)** αvβ6 mediated migration. (Left) Transwells were coated with either fibronectin (Fn) or LAP, and 50,000 C76 cells seeded in top of the transwell, either treated with 264RAD or an IGG control. Treatment with 264RAD caused a significant decrease in migration towards LAP but not Fn. Data represents mean ± SD (*n* = 3). (Right) Transwells were coated with 0.5 μg/ml of LAP and added to the top of the transwell in the presence of chlorpromazine, filipin, 264RAD or vehicle control. Significant reductions in the number of cells migrating through the transwells were observed with chlorpromazine, filipin and 264RAD.

To establish the functional effects of impeding αvβ6 endocytosis *in vitro*, migration assays were performed towards latency associated peptide (LAP) the high affinity ligand for αvβ6. Firstly, LAP internalisation in C76 cells was confirmed (Figure 4A). Using flow cytometry and an Fc-tagged LAP peptide (generous gift from Shelia Violette at Biogen Idec), 83 ± 10% of LAP was internalised by C76 cells in 60 min. The rate and total amount of endocytosis was not significantly different to Cy5-SS-(GS)_5_-bioA20FMDV2 internalisation suggesting the two ligands were considered very similar by αvβ6. Internalisation of LAP-Fc was also confirmed by immunofluorescent imaging (Figure 4A).

To confirm that migration toward LAP was αvβ6 specific, migration towards fibronectin and LAP was quantified with and without antibody-blockade of αvβ6 with 264RAD (Figure 4B). Relative migration (normalised to uncoated, 10% FBS control), was 0.8 ± 0.1 in fibronectin coated Transwells^©^, which decreased to 0.5 ± 0.2 when treated with 264RAD (*n* = 3, ns). Relative migration towards LAP was 0.8 ± 0.3, and 0.2 ± 0.2 in the 264RAD treated cells (*n* = 3, *p* < 0.05). Thus, migration of C76 cells towards LAP was quantified in the presence of chlorpromazine and filipin. The number of vehicle control-treated cells migrating towards LAP was 547 ± 128 whereas in the presence of chlorpromazine, filipin and 264RAD the number of migrating cells was 225 ± 73, 280 ± 51 and 115 ± 34, respectively); each of these reductions were statistically significant (students paired *t*-test, *p* < 0.05). These data suggest that the αvβ6 specific migration towards LAP is impeded by inhibition of clathrin or caveolin mediated endocytosis.

### Subcellular location of αvβ6 post-endocytosis

**FIGURE 5:**
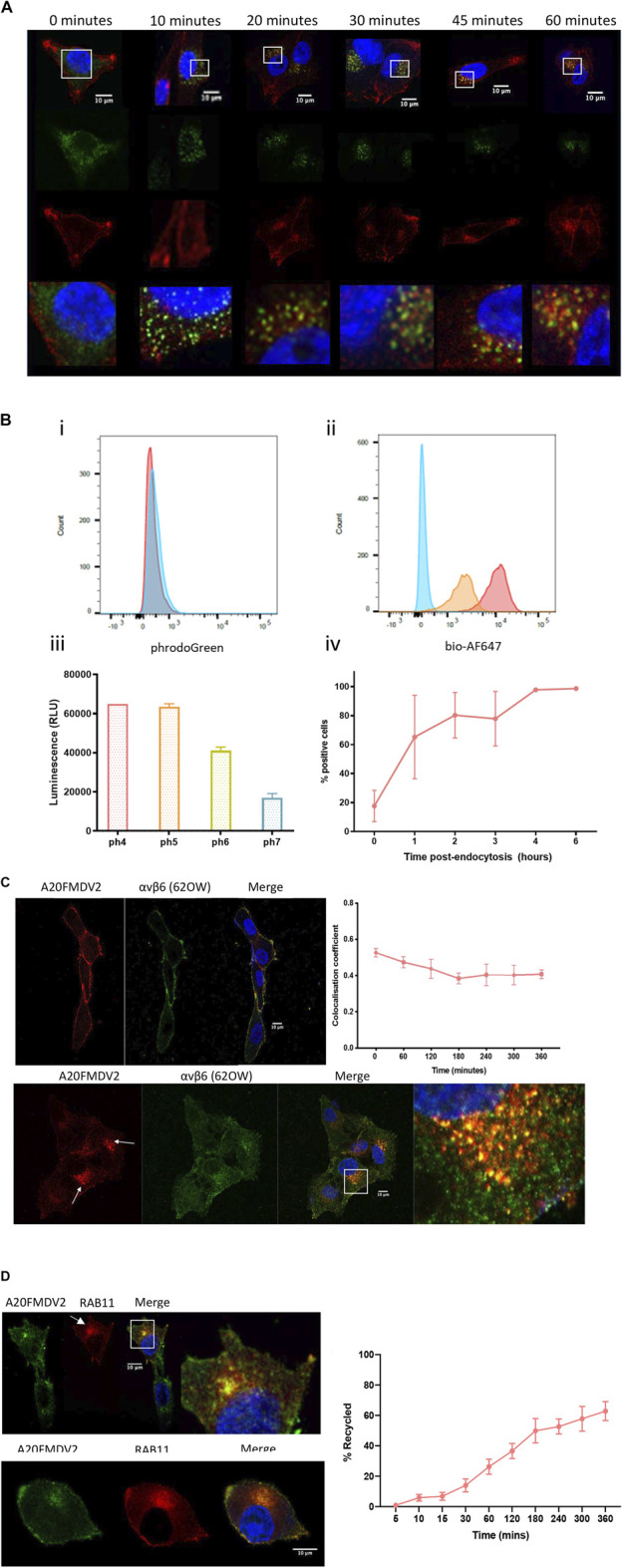
(Continued).

Cy5-(GS)_5-_bioA20FMDV2 peptide was used to determine the subcellular location of αvβ6 in cells up to 360 min post-endocytosis. Firstly, following internalisation of Cy5-(GS)_5-_bioA20FMDV2, the A375PB6 cells were fixed and stained with early endosome marker EEA1 (representative images in Figure 5A). Cy5-(GS)_5-_bioA20FMDV2 was internalised into cells from 10 min and was observed from 20 min accumulating in perinuclear compartments, which were observed in each time point thereafter up to 1-h post-endocytosis. Colocalisation analysis of EEA1 and A20FMDV2 revealed a significant increase in A20FMDV2 and EEA1 colocalisation from 0 to 10 min (0.19 ± 0.09 to 0.39 ± 0.13, *p* < 0.0001) (*n* = 54), and from 10 to 20 min (0.49 ± 0.14, *p* < 0.001). Cy5-(GS)_5-_bioA20FMDV2 remained in perinuclear clusters from 1 to 6 h, however these clusters were no longer EEA1 positive (Figure 5A).

The location of endosomal cargo can also be estimated using a range fluorochromes sensitive to pH changes. One such fluorochrome is pHrodo™, which at a neutral pH emits limited fluorescence, but in an acidic environment fluoresces brightly. This fluorochrome was conjugated as per manufacturer’s instructions to a biotinylated A20FMDV2, and fluorescence measured following internalisation by flow cytometry. A20FMDV2-pHrodo retained specificity to αvβ6 and demonstrated pH sensitivity (Figure 5B). Fluorescence intensity of A20FMDV2-pHrodo increased following internalisation from 0 h (17.6 ± 10.8%) to 4 h (97 ± 1.1%, *n* = 3) and remained stable between 4 and 6 h (Figure 5.4B).

To confirm that Cy5-(GS)_5_-bioA20FMDV2 and αvβ6 remained associated during the 6-h time frame, αvβ6 was labelled with both Cy5-(GS)_5-_bioA20FMDV2 and 62OW (non-ligand mimetic αvβ6 specific antibody) (Figure 5C). Both Cy5-(GS)_5-_bioA20FMDV2 (red) and 62OW (green) can be seen at the surface of the cells at the 0-time point, with a mean colocalisation coefficient of 0.52 ± 0.04 (±SD, *n* = 3). Again, A20FMDV2 clustered to perinuclear compartments (Figure 5C) as was previously described above following internalisation. While the 62OW staining is more dispersed throughout the cell following internalisation, the perinuclear clusters show both A20FMDV2 and 620W staining, and colocalization showed no significant decrease from time point 0 h up to 6 h post internalisation, suggesting A20FMDV2 remains bound to αvβ6 in the peri-nuclear compartment.

### Ligand-bound αvβ6 is recycled

In the siRNA screen, PPFIA1, when knocked down, significantly increased the amount of αvβ6 internalised into the cell by 60 ± 11% (*p* < 0.05) (Figure 3). The observation that knockdown of a gene could cause an increase in endocytosis led us to consider what could be the mechanism. One hypothesis was that integrin recycling was being reduced, thus causing an accumulation of αvβ6-bound Cy5-SS-(GS)_5_-bioA20FMDV2 inside the cell, which would usually be recycled.

To establish if ligand-bound αvβ6 is recycled, the association of αvβ6 with Rab11, a marker of long-loop recycling (Roberts et al., 2001; Powelka et al., 2004; Strachan and Condic, 2004; Skalski and Coppolino, 2005; Das et al., 2018; Howe et al., 2020), was determined within the same 6-h time frame as previously studied (Figure 5D). Immunofluorescent images of A375β6 cells expressing Rab11-RFP revealed colocalisation of Rab11 with Cy5-(GS)_5_-bioA20FMDV2 from 1 h post endocytosis. Thus, the flow cytometry-based internalisation assay was modified to establish whether ligand-bound αvβ6 was recycled back to the cell surface. In preliminary studies we confirmed that the reducing agent TCEP very efficiently removed the Cy5 from Cy5-SS-(GS)_5_-bioA20FMDV2 bound to the surface of cells at 4C in the presence of 0.1% NaN_3_ (Supplementary Figure 3). Next, cells exposed to Cy5-SS-(GS)_5_-bioA20FMDV2 for 20 min were treated with TCEP then returned to 37°C. At time points thereafter the cells were re-exposed to TCEP and the difference in fluorescence used to calculate the fraction of ligand-bound integrin returning to the surface.

Using this model, it was revealed that 62% (±6.2%) of the αvβ6-A20FMDV2 internalised after 20 min was recycled back to the membrane over the course of 6 h (Figure 5D). The highest rate of recycling was observed between 30 and 180 min (from 13 ± 4.2% to 49 ± 8%). After 3 h of recycling, the rate slowed, and only a further 13% of internalised peptide was recycled over the remaining 3 h (49 ± 8% after 3 h to 62 ± 6.2% after 6 h).

### αvβ6 degradation

**FIGURE 6:**
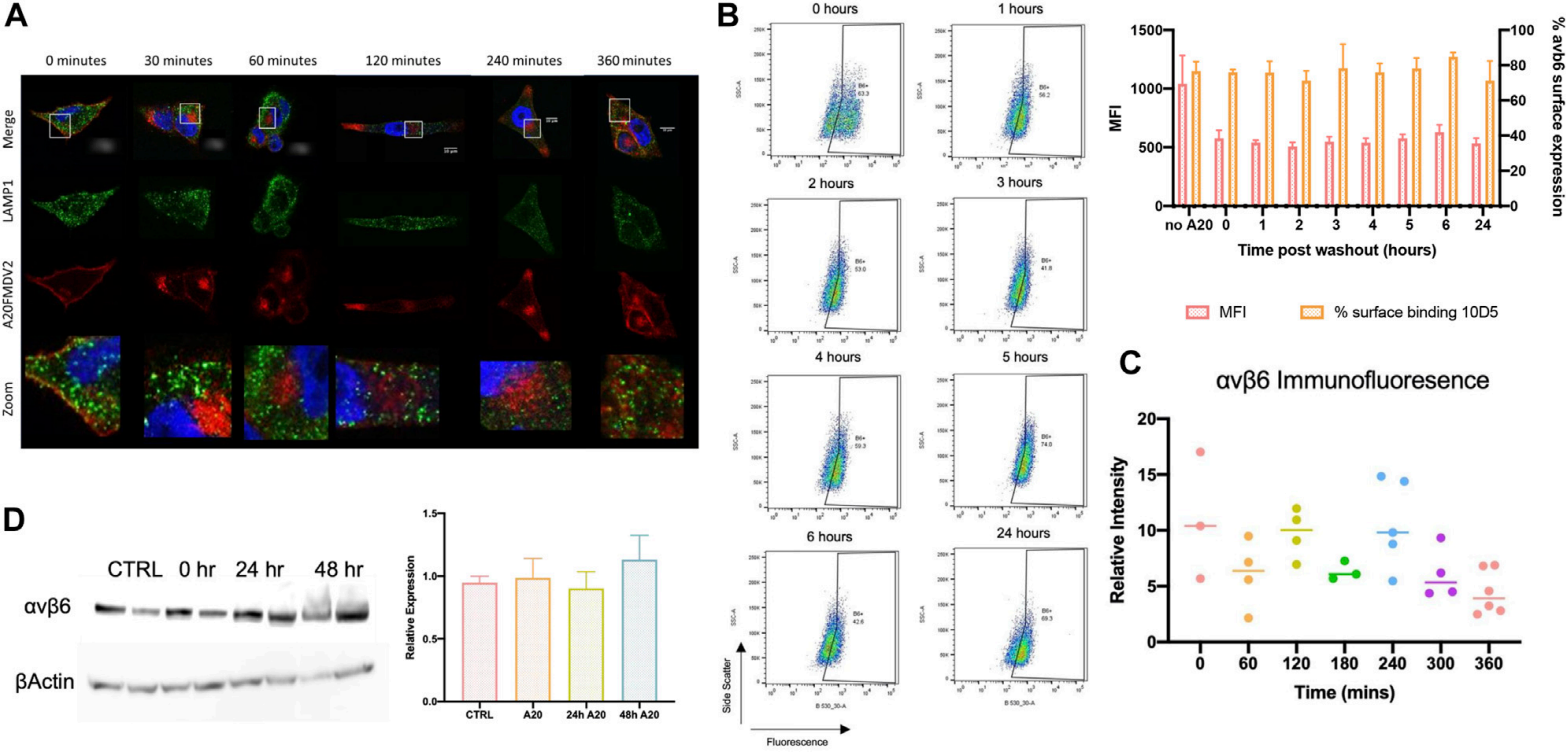
αvβ6 degradation post-endocytosis. **(A)** Representative images show the distribution of A20FMDV2 (red) and LAMP1 (green) in A375β6 cells following Cy5-(GS)5-bioA20FMDV2 internalisation. LAMP1 staining is consistent in each time point, with no indication of changes over time (up to 360 min). **(B)** αvβ6 surface expression was monitored using 10D5 by flow cytometry (example dot-plots and summary histogram shown) post-exposure to A20FMDV2 up to 24 h after wash-out, with no significant differences observed in expression over this time course in the percentage of cells bound to αvβ6. There was a significant reduction in MFI of 10D5 binding with and without A20FMDV2 pretreatment (*n* = 3). **(C)** Quantification of 62OW staining intensity from images acquired in Figure 5, revealing no significance in the intensity up to 6 h post A20FMDV2 exposure. **(D)** αvβ6 expression was also quantified by western blotting for the β6 subunit, with no changes in expression up to 48 h post-exposure to the ligand. Data represents mean ± SD, *n* = 3. Blot features two technical repeats per condition.

LAMP1 is a glycoprotein expressed on the membrane of lysosomes, and therefore acts as a selective indicator of these endosomes (Balderhaar and Ungermann, 2013). The degree of LAMP1 colocalisation with Cy5-(GS)_5-_bioA20FMDV2 prior to internalisation was 0.22 ± 0.1 (*n* = 4) (Figure 6A). No significant differences in colocalisation of LAMP1 and Cy5-(GS)_5_-bioA20FMDV2 were observed at any time point post- internalisation (2 h 0.27 ± 0.03, 3 h 0.20 ± 0.08, 4 h 0.20 ± 0.07, 5 h 0.21 ± 0.09, 6 h 0.19 ± 0.08), suggesting that no trafficking of A20FMDV2 in to late endosomes occurred during this time frame. Furthermore, expression of αvβ6 protein was determined using antibody 10D5 after exposure to Cy5-(GS)_5_-bioA20FMDV2 (Figure 6B and 6C). 10D5 is still able to bind to αvβ6 since a non-saturating concentration of A20FMDV2 (100 nm) is used Firstly, surface expression was measured by flow cytometry, revealing no significant differences across any time point studied (76 ± 2% at 0 min, 71 ± 15% after 24 h) when considering the percentage of cells expressing αvβ6 (*n* = 3). However there was a difference observed in the mean fluorescence intensity (MFI) of the amount of bound 10D5 pre and post A20FMDV2 treatment, since fewer αvβ6 complexes are available, but this is unchanged from 1 to 24 h post treatment. Secondly, the intensity of the 62OW staining was determined and normalised to the area of the cell measured. While small changes in the fluorescence intensity were observed (0 min 11 ± 5.5, 60 min 6 ± 3, 120 min 9.7 ± 2.2, 180 min 6.3 ± 0.8, 240 min 10.6 ± 3.9, 300 min 6.1 ± 2.3 and 360 min 4.4 ± 1.9), no significant differences were observed between any time point. Finally, total αvβ6 expression was analysed using western blotting for the β6 subunit (Figure 6D). Once again, no differences were observed in αvβ6 expression following exposure of cells to Cy5-(GS)_5_-bioA20FMDV2. Quantification of the western blots revealed relative expression of 0.97 ± 0.2 at time point 0, 0.8 ± 0.2 after 24 h and 1.1 ± 0.2 after 28 h (*n* = 3, ns).

## Discussion

ImageStream flow cytometry, immunofluorescent staining and flow cytometry assays showed that the αvβ6-specific fluorescently labelled A20FMDV2 variants (Cy5-(GS)_5_-bioA20FMDV2 and Cy5-SS-(GS)_5_-bioA20FMDV2) bound to αvβ6 was internalised by multiple cell lines from different tissue types to a similar degree (Figure 2). The data from both flow cytometry-based methods revealed that the majority of αvβ6 was internalised in the first 20 min, with full internalisation occurring within 60 min. This rate of internalisation was similar to previously published data (Saha, 2011; Slack et al., 2016).

An siRNA screen revealed that fifteen genes significantly reduced the rate of αvβ6 – Cy5-SS-(GS)_5_-bioA20FMDV2 endocytosis into C76 pancreatic cancer cells (Figure 3). Of these fifteen genes, nine play a role in clathrin-mediated endocytosis, while 5 play a role in caveolin-regulated endocytosis, both of which are endocytic pathways regulated by dynamin (Nabi and Le, 2003; Stang et al., 2004; Takei et al., 2005; Doherty and McMahon, 2009; Marina-García et al., 2009; Naslavsky and Caplan, 2011). Additionally, five genes play a role in dynamin independent pathways, although, when these genes were knocked down and drug inhibition of clathrin performed, no further reduction in the rate of endocytosis was observed; these data suggest that the potential dynamin-independent activities of these five genes (CBL, CBLB, CYTH2, ARFGAP and ASAP) did not affect endocytosis of αvβ6-bound to A20FMDV2.

The role of clathrin in αvβ6 mediated endocytosis has previously been described (Berryman et al., 2005; Ramsay et al., 2007; John et al., 2020). This was first suggested by Berryman et al. (2005) who showed that clathrin inhibition by non-specific sucrose treatment reduced αvβ6 dependent FMDV infection by 95%. Thereafter, Ramsay et al. (2007) demonstrated a ∼40% reduction in αvβ6 endocytosis using CLTC siRNA (Ramsay et al., 2007), while John et al. (2020) showed a similar rate of reduction in ligand induced endocytosis using a clathrin-specific pharmacological inhibitor (John et al., 2020). The differences in levels of endocytosis reported by these authors is most likely due to the different methods used, especially the sucrose method as it is the least specific, and has previously been shown to inhibit other endocytosis pathways (Guo et al., 2015). Additionally, Ramsay et al. (2007) determined that HAX-1, which binds to the β6 cytoplasmic tail, when knocked down, significantly reduces αvβ6 endocytosis. While HAX-1 was not a statistically significant hit in this screen, there was still an overall reduction (42 ± 16%) in the amount of αvβ6 endocytosed in HAX-1 knockdown cells compared to the control condition (Figure 3A).

While the role of clathrin-mediated αvβ6 endocytosis had previously been established, the role of caveolin-dependent mechanisms in this process was not certain. Although Berryman et al. (2005) stained for caveolin-positive lipid rafts post endocytosis of FMDV-bound to αvβ6, they did not detect any colocalization with αvβ6, later showing that the cell lines used (SW480) did not express caveolin-1. John et al. (2020) specifically inhibited caveolin carriers using filipin, a small molecule inhibitor of caveolin (Schnitzer et al., 1994). While a reduction in internalisation with this inhibitor was observed, this was not statistically significant. In contrast, in this study, all three caveolin genes, when knocked down independently, resulted in a significant reduction in αvβ6 endocytosis (Figures 3A,B).

One gene, PPFIA1, when knocked down by siRNA, significantly increased the amount of αvβ6 internalised into the cell by 60 ± 11% (*p* < 0.05) (Figure 3). PTPRF Interacting Protein Alpha 1 (PPFIA1), also known as Liprin α1, is a cytoplasmic protein known to be involved in regulation of cell migration and cell spreading (Shen et al., 2007). In cancer, it has been shown to regulate invasion, motility, and extracellular matrix degradation (Astro et al., 2011). Specifically, Liprin α1-mediated cell spreading has been shown to be dependent on the distribution and trafficking of activated integrins (Asperti et al., 2009). Asperti et al. (2009) showed that when Liprin α1 is depleted, cell spreading is reduced on collagen due to an inability to form focal adhesions, and that this mechanism is dependent on the interaction of Liprin α1 with talin.

Although PPFIA1 was the only gene from our screen to significantly increase the rate of endocytosis, there were many others which increased the rate that did not achieve significance, some of which are known to regulate recycling (e.g., RUFY1, Yamamoto et al., 2010). This supports the hypothesis that the PPFIA1 effect may be due to an inhibition of recycling as opposed to an actual increase in endocytosis. In 2016, Mana et al., identified that PPFIA1 was involved in the recycling of fibronectin-bound α5β1 (Mana et al., 2016). They showed that post-endocytosis, α5β1 remained bound to fibronectin and recycled via a post-golgi compartment back to the cell membrane. In the absence of PPFIA1, which interacts with the integrin cytoplasmic tail, α5β1 accumulated in the post-golgi compartments, unable to recycle, causing the cell to lose its polarity. Crucially, inhibition of clathrin and caveolin significantly impacted migration of cells towards αvβ6 specific ligand LAP (Figure 4).

In the first 20 min of endocytosis, αvβ6-A20FMDV2 is significantly colocalised with early endosome marker EEA1 (Figure 5), a Rab5 effector protein (Wilson et al., 2000), and an antigen ubiquitously used to identify early endosomes (Lee et al., 2020; Vandesande et al., 2020; Eapen et al., 2021; Holst et al., 2021; Wang et al., 2021). A significant shift in colocalization of A20FMDV2 (Cy5-(GS)_5_-bioA20FMDV2) with EEA1 endosomes from 1 to 2 h (*p* < 0.001) was also observed. LAMP1, a late endosome marker showed no differences in colocalisation up to 6 h post-internalisation. It was originally hypothesised that at least a fraction, or possibly a significant amount of αvβ6 would be degraded; John et al., 2020 demonstrated using an αvβ6 specific antibody that αvβ6 expression was lost 1 h post endocytosis of the integrin and its ligand (John et al., 2020). Additionally, other integrins have been identified in late endosomes/lysosomes post-internalisation within this time frame; in particular ligand occupied and active integrin (Dozynkiewicz et al., 2012).

Internalisation assays performed with the pHrodo conjugated A20FMDV2, revealed that A20FMDV2-pHrodo-αvβ6 is trafficked continually into a more acidic environment up to 6 h after internalisation, with the biggest shift in fluorescence observed in the first 2 h post-endocytosis, suggesting that, despite the absence of A20FMDV2 in late endosomes, that αvβ6-bound A20FMDV2 continues to be trafficked for this relatively long-time frame.

Post-endocytosis, integrins are either recycled or degraded. Recycling occurs, as previously discussed, via Rab4 (short) or Rab11 (long) mediated recycling (Bridgewater et al., 2012; Goldenring, 2015). Given that Cy5-(GS)_5-_bioA20FMDV2 -αvβ6 is still present within the cells 6 h post-internalisation, the role of recycling was investigated. So called “long-loop” recycling is mediated by Rab11, and it is established that many integrins recycle via Rab11 positive compartments including β1 integrins (Roberts et al., 2001; Powelka et al., 2004; Strachan and Condic, 2004; Skalski and Coppolino, 2005; Das et al., 2018; Howe et al., 2020). Specifically, intracellular integrins have been found in Rab11 subcellular compartments within 1 h of endocytosis (Das et al., 2018; Howe et al., 2020). Powelka et al. (2004) demonstrated that β1 is internalised to perinuclear compartments 30 min (Powelka et al., 2004), and subsequently showed that these compartments are Rab11 positive, and present in the cells up to 2 h post internalisation.

Immunofluorescent staining of Rab11-RFP transfected cells revealed that A20FMDV2-αvβ6 is present in Rab11 positive compartments in the cell, including at the cell surface, from 1-h post-endocytosis for at least 6 h (Figure 5D). To confirm the role of αvβ6 recycling, the return of αvβ6–A20FMDV2 back to the cell surface following internalisation was measured using flow cytometry (Figure 5). Data showed that ligand-bound αvβ6 is steadily recycled with 62% of the peptide internalised within 20 min recycled over 6 h. Interestingly, we initially considered that the natural reducing environment of the cytoplasm may result in reduction of the di-sulphide bond of the integrin bound Cy5-SS-(GS)_5_-bioA20FMDV2. However, the data showed clearly that a TCEP-sensitive fluorescent pool of peptide re-appeared on the surface after re-incubation at 37°C. In support of our conclusions, using immunofluorescence analysis, in Figures 5C,D, Cy5-(GS)_5_-bioA20FMDV2 colocalises at the cell surface with 620W, and Rab11, respectively.

The implications of ligand-bound αvβ6 recycling are multiple. Firstly, we must consider the implications in the context of known αvβ6 functions. Arguably, the most important function of αvβ6 is to activate TGFβ by binding LAP (Munger et al., 1999). Hypothetically, if LAP bound to αvβ6 was internalised and recycled back to the cell surface, this would not allow for further activation of TGFβ, as the αvβ6 ligand-binding site would remain occupied. This would ultimately mean that the cells would become unable, or have a reduced propensity, to activate TGFβ; a process which has been previously observed by Vizán et al. (2013) (Vizán et al., 2013). Secondly, recycling of ligand-bound αvβ6 would also affect how αvβ6 is therapeutically targeted. As previously mentioned, small molecule inhibitors targeting αvβ6 are currently under development (Slack et al., 2016; John et al., 2020), the fate of these small molecule inhibitors post uptake into the cell is critical for determining optimal administration and dosage.

Finally, we must also consider possible unknown functions of αvβ6 in relation to recycling. For example, β1 integrin recycling is critical for sustaining polarity of fibroblasts during migration (Jacquemet et al., 2013; Samarelli et al., 2020). Recycling of α5β1 active integrins is also hypothesised to play a role in the turnover of fibronectin, whereby the cleaved fibronectin from the ECM is exchanged in the trans-Golgi network for a newly synthesised fibronectin molecule, which is subsequently returned to the membrane at the basolateral cell surface, also contributing of maintenance of cell polarity (Mana et al., 2016; Mana et al., 2020).

No such role for αvβ6 recycling has been identified; however, based on evidence from other integrins that they continue to interact with proteins from endosomal compartments, it’s reasonable to speculate that αvβ6 trafficking may also contribute to intracellular signalling. These could include regulation of cell migration, invasion and/or proliferation, all established functions of αvβ6 (Huang et al., 1998; Thomas et al., 2001a; Thomas et al., 2001b; Ahmed et al., 2002; Reader et al., 2019).

The presence of αvβ6 6-h post internalisation is in contrast to what has been previously published by John et al. (2020), who showed that αvβ6 was degraded post internalisation of an αvβ6 -specific ligand in to NHBE cells. The extent of potential αvβ6 degradation was assessed in these studies by measuring the intensity of the fluorescence of mAb 62OW at each time point, by both western blotting and flow cytometry, revealing no significant differences in expression of αvβ6 over time (Figure 6). We are unable to explain the difference in our results compared with the study by John et al.

Post-endocytosis events of FMDV by αvβ6 previously have been studied (Berryman et al., 2005). Berryman and others showed that FMDV colocalised with early endosomes up to 30 min post entry and did not find any colocalisation with late endosome marker LAMP2 within this same time frame. While these data are consistent with the data presented here, it fails to explain how αvβ6 expression is depleted post-internalisation, without trafficking to a late endosome or lysosomal compartment. It must be noted that the work performed by John et al. (2020) was using normal human bronchial epithelial (NHBE) cells, and thus we considered it could be a difference in behaviour observed in cancer cells compared with normal epithelial cells. However, immunofluorescent staining for αvβ6 post-exposure to A20FMDV2 (Cy5-(GS)_5-_bioA20FMDV2) was repeated in NHBE cells, and again we found no difference in expression of αvβ6 (Supplementary Figure 2).

**FIGURE 7:**
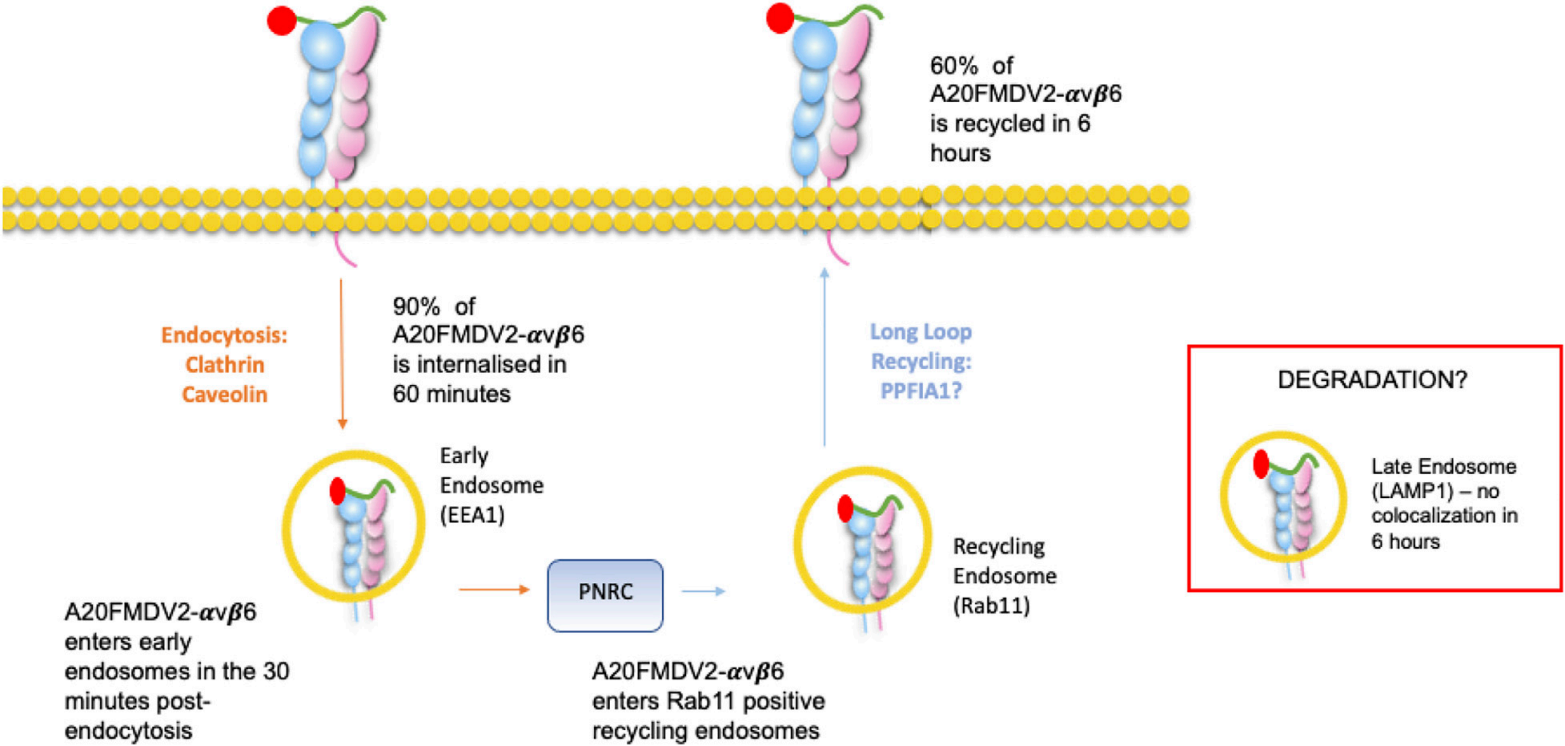
Ligand-bound endocytosis and trafficking of αvβ6 Summary figure for this study. A20FMDV2-αvβ6 internalises by clathrin and caveolin mediated endocytosis into early endosomes, where it colocalises with EEA1 in the first 30 min post endocytosis. From the early endosome, A20FMDV2-αvβ6 accumulates in perinuclear clusters, which are positive for Rab11 (1–6 h post endocytosis). Within this 6-h time frame, 60% of internalised A20FMDV2-αvβ6 is recycled back to the cell surface.

In summary, as described in Figure 7, ligand (A20FMDV2 or LAP)-induced endocytosis of αvβ6 is a combination of both caveolin and clathrin-mediated processes whereby over 90% is internalised within 60′. The majority of the A20FMDV2-bound endocytosed αvβ6 passes first through EEA1 early endosomes before forming a pool of Rab11-positive acidified perinuclear endosomes. Finally, 60% of this internalised αvβ6 recycles back to the cell surface within 6 h, still bound to the A20FMDV2 ligand. These data should be considered by those developing high affinity αvβ6-ligand mimetic targeting as it could determine the success or failure of repeat dosing of therapeutics.

## Data Availability

The original contributions presented in the study are included in the article/Supplementary Material, further inquiries can be directed to the corresponding author.

## Supplementary material

Supplementary Figure 1 αvβ6 internalisation in adherent vs. suspension cells. Cells were exposed to A20FMDV2 for 1 h on ice. Unbound peptide was washed away by either centrifugation (suspension cells) or by washing the wells in DMEM 0.1/0.1. Cells were incubated at 37°C for 1 h to allow for internalisation, and subsequently acid stripped as previously described. Cells were acquired by flow cytometry as described in materials and methods. No significant differences were observed between the internalisation between cells in suspension vs adhered (*n* = 2).

Supplementary Figure 2 αvβ6 expression (green) in NHBE cells before and after treatment with A20FMDV2.

Supplementary Figure 3 TCEP concentration optimisations. C76 cells were exposed to Cy5-SS-(GS)5-bioA20FMDV2, and extracellular fluorescence removed by addition of varying concentrations of TCEP (50, 100, 250, 500 mM). A reduction in both the number of positive cells and relative geometric mean fluorescence of the cells were observed with each concentration (1.9%, 1.4%, 1.0%, 0%, and 1.38, 1.24, 1.69, 1.61, respectively) compared to control conditions (97% and 14.43). Viability was determined by flow cytometry and the uptake of DAPI in to compromised cells. The lowest cell viability was observed with 500nM of TCEP, with only 68% of cells viable, compared with 95% of cells without TCEP [mean values (*n* = 2)].

Supplementary Figure 4 Western Blot confirming knockdown of Clathrin (CLTC) and Caveolin (CAV) Knockdown with siRNA. Relative expression of CLTC in the off target (OT) transfected cells was 0.79 ± 0.2 and 0.29 ± 0.23 in cells transfected with the CLTC smartpool. Relative expression of CAV in the off target (OT) transfected cells was 0.79 ± 0.05 and 0.23 ± 0.28 in cells transfected with the CLTC smartpool. Data are mean ± SD, representative of two biological repeats each with two technical repeats. Representative western blots are shown.

Supplementary Table 1 αvβ6 expression of cell lines used, determined by binding of A20FMDV2 (*n* = 3).
