# Supplementary material for: Ligand-bound integrin αvβ6 internalisation and trafficking
Source: Front Cell Dev Biol. 2022 Aug 24;10:920303. doi: 10.3389/fcell.2022.920303 (PMC9448872; doi:10.3389/fcell.2022.920303)
Supplement: Supplementary file 1 [file Presentation1.pdf]

Supplementary Figures

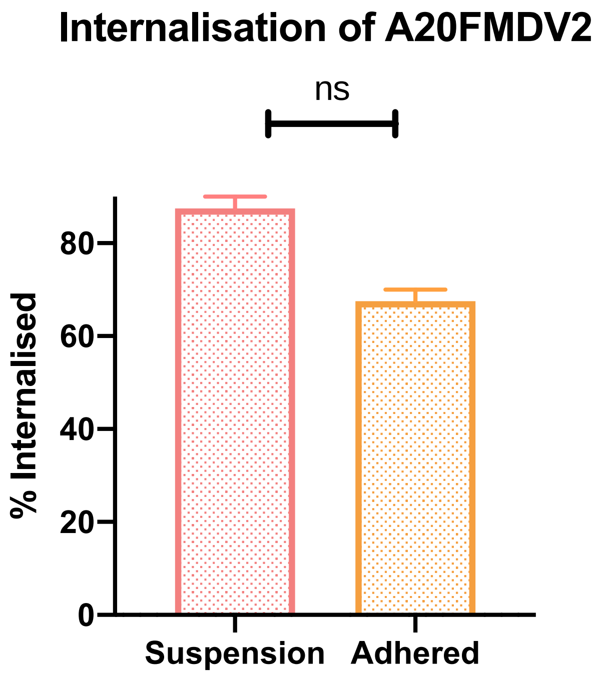


**Supplementary Figure 1 αvβ6 internalisation in adherent vs suspension cells.** Cells were exposed to A20FMDV2 for 1 hour on ice. Unbound peptide was washed away by either centrifugation (suspension cells) or by washing the wells in DMEM 0.1/0.1. Cells were incubated at 37°C for 1 hour to allow for internalisation, and subsequently acid stripped as previously described. Cells were acquired by flow cytometry as described in materials and methods. No significant differences were observed between the internalisation between cells in suspension vs adhered. (n=2)

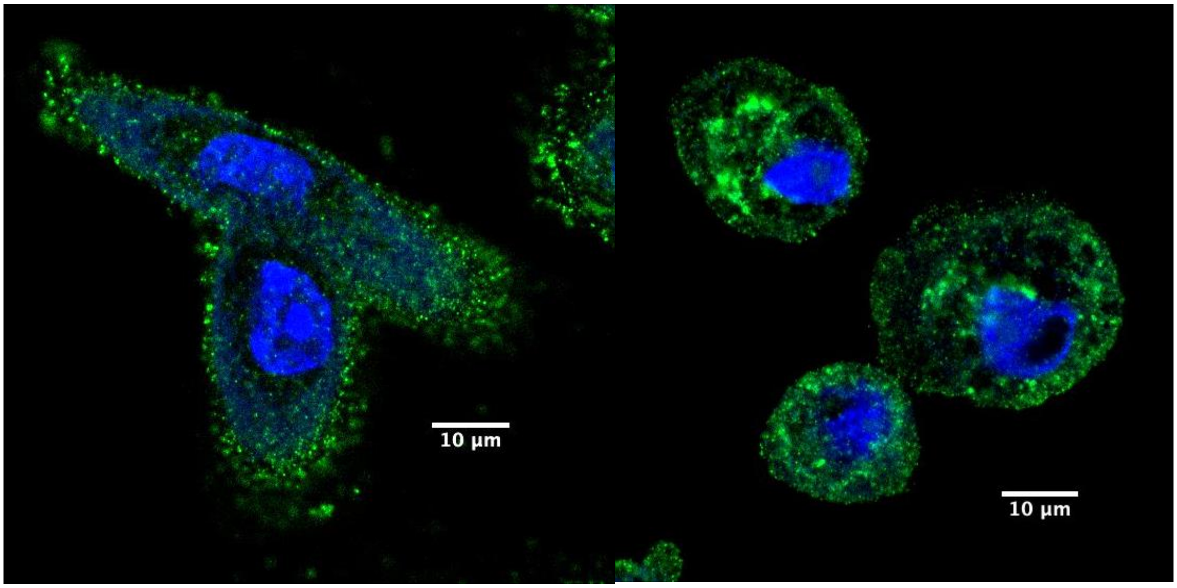


0 hours 24 hours

**Supplementary Figure 2** αvβ6 expression (green) in NHBE cells before and after treatment with A20FMDV2

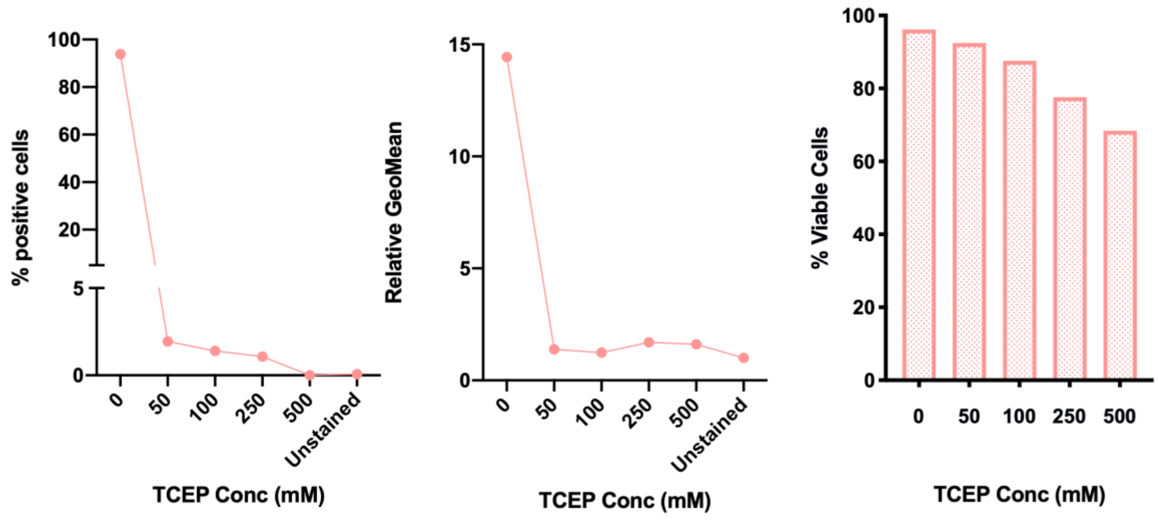


**Supplementary Figure 3. TCEP concentration optimisations.** C76 cells were exposed to Cy5-SS-(GS)5-bioA20FMDV2, and extracellular fluorescence removed by addition of varying concentrations of TCEP (50, 100, 250, 500mM). A reduction in both the number of positive cells and relative geometric mean fluorescence of the cells were observed with each concentration (1.9%,1.4%,1.0%,0% and 1.38, 1.24, 1.69, 1.61, respectively) compared to control conditions (97% and 14.43). Viability was determined by flow cytometry and the uptake of DAPI in to compromised cells. The lowest cell viability was observed with 500nM of TCEP, with only 68% of cells viable, compared with 95% of cells without TCEP (mean values, (n=2)).

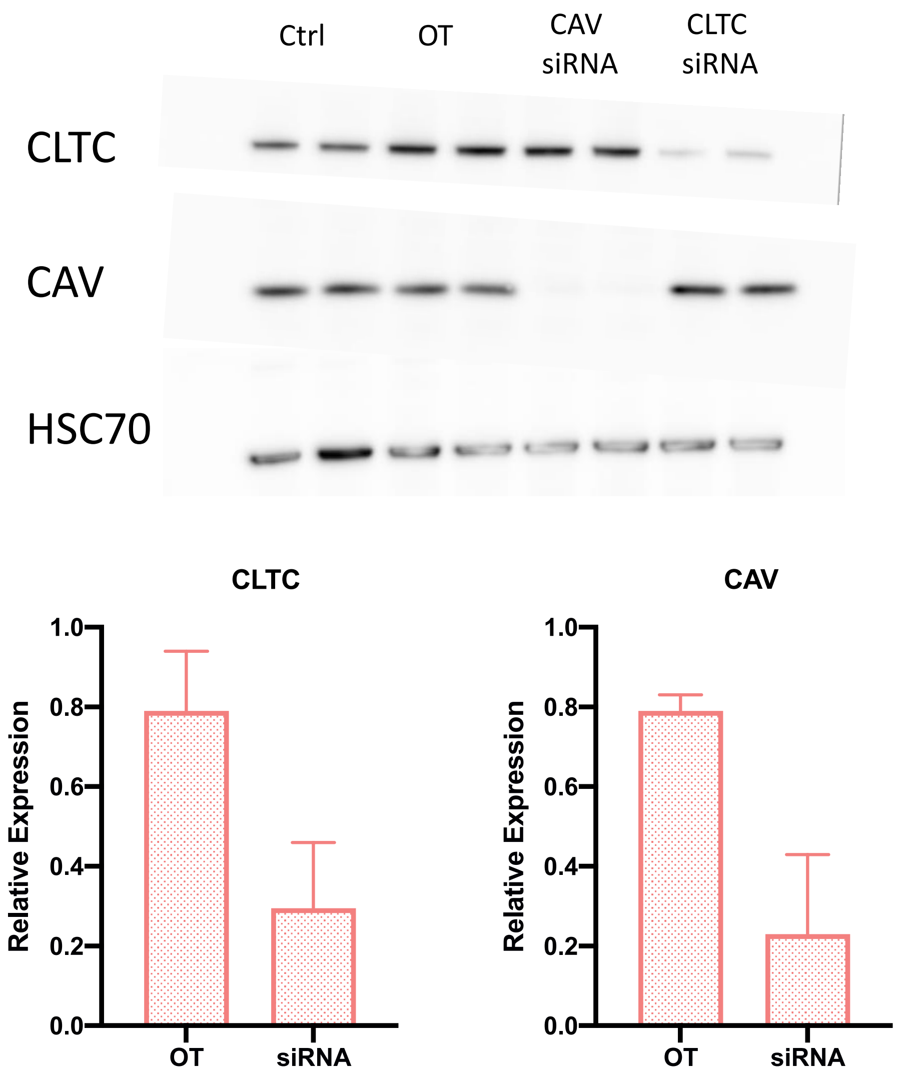


**Supplementary Figure 4. Western Blot confirming knockdown of Clathrin (CLTC) and Caveolin (CAV) Knockdown with siRNA.** Relative expression of CLTC in the off target (OT) transfected cells was 0.79+/- 0.2 and 0.29 +/- 0.23 in cells transfected with the CLTC smartpool. Relative expression of CAV in the off target (OT) transfected cells was 0.79+/- 0.05 and 0.23 +/- 0.28 in cells transfected with the CLTC smartpool Data are mean +/- SD, representative of 2 biological repeats each with 2 technical repeats. Representative western blots are shown.

| Cell Line | Mean | SD |
|---|---|---|
| C76 | 99.13 | 0.72 |
| C102 | 99.67 | 0.32 |
| C139 | 99.93 | 0.06 |
| H322M | 99.77 | 0.15 |
| H441 | 99.67 | 0.21 |
| MCF10A | 16.17 | 16.44 |
| MCF10 CA1alpha | 100.00 | 0.00 |
| MDAMB468 | 98.30 | 1.84 |
| HPDE | 83.30 | 26.52 |

**Supplementary Table 1. αvβ6 expression of cell lines used, determined by binding of A20FMDV2 (n=3)**
